# Pathological Stage and Grade Determine the Impact of Focal Versus Extensive Positive Surgical Margins After Radical Prostatectomy

**DOI:** 10.3390/cancers18071123

**Published:** 2026-03-31

**Authors:** Marco Oderda, Daniele Calvo, Giorgio Calleris, Giuseppe Carlo Iorio, Alessandro Marquis, Giancarlo Marra, Umberto Merani, Alberto Sasia, Alessio Venturi, Paolo Gontero

**Affiliations:** 1 Division of Urology, Department of Surgical Sciences, Molinette Hospital, University of Turin, 10126 Turin, Italy; daniele.calvo@unito.it (D.C.); giorgio.calleris@unito.it (G.C.); alessandro.marquis@unito.it (A.M.); giancarlo.marra@unito.it (G.M.); umbertomaria.merani@unito.it (U.M.); alberto.sasia@unito.it (A.S.); alessio.venturi@unito.it (A.V.); paolo.gontero@unito.it (P.G.); 2 Division of Radiation Oncology, Department of Oncology, Molinette Hospital, University of Turin, 10126 Turin, Italy; giorio@cittadellasalute.to.it

**Keywords:** prostate cancer, positive surgical margins, stage, grade, biochemical recurrence

## Abstract

**Purpose**: the prognostic value of positive surgical margins (PSMs) after radical prostatectomy (RP) remains debated, particularly when distinguishing focal from extensive involvement. Moreover, the interaction between margin status and other adverse pathological features, such as tumour stage, grade, and nodal status, is not fully defined. The study aims to evaluate the impact of focal and extensive PSMs on biochemical recurrence-free survival (BCRFS), stratified by pathological stage, Gleason grade, and nodal involvement. **Methods**: we retrospectively analyzed 1258 patients who underwent robot-assisted RP between 2017 and 2023, evaluating BCRFS as primary endpoint. Survival was analyzed using Kaplan–Meier and log-rank tests across margin categories (negative, focal, extensive). Analyses were stratified by pT stage (pT2–pT3b), ISUP grade (<3 vs. ≥3), and nodal status (pN0/pNx vs. pN+). Median follow-up was 39 months (IQR 24–62), and 5-year BCRFS was used as the reference endpoint. **Results**: median age was 68 years and median PSA 6.9 ng/mL. Stage distribution: pT2 56.4%, pT3a 29.8%, pT3b 13.6%; 16.6% were pN+. ISUP grade ≥ 3 was observed in 60.6%. Overall, 20.3% developed biochemical recurrence. In pT2 tumours or primary Gleason pattern 3, focal PSMs showed 5-year BCRFS of 88–90%, very similar to negative margins (91–93%) and clearly better than extensive PSMs (~72%). In pT3 tumours or primary Gleason pattern 4, focal PSMs showed 5-year BCRFS of ~55–60%, similar to extensive PSMs (~40–50%) and significantly worse than negative margins (78% in pT3a and 65% in pT3b, *p* < 0.01). In N + 5-year BCRFS was similarly poor in negative (~45%), focal (~42%), and extensive margins (~40%, *p* = 0.455). **Conclusions**: the prognostic impact of positive surgical margins depends on tumour biology. In organ-confined or ISUP ≤ 2 disease, focal PSMs show recurrence rates similar to negative margins and may be managed with observation. In locally advanced or ISUP ≥ 3 tumours, both focal and extensive PSMs carry a markedly higher risk of biochemical recurrence and might suggest an early intervention. PSMs lose discriminatory value in node-positive patients.

## Simple Summary

In this single-center retrospective series of 1258 robot-assisted radical prostatectomies (RARPs), the biochemical recurrence rate was 20.3% after a median follow-up of 39 months. The prognostic impact of positive surgical margins (PSMs) appeared to be modulated by tumor biology. In organ-confined disease and in tumors with ISUP grade ≤ 2, focal PSMs were associated with recurrence rates comparable to those of negative margins, suggesting that observation may be a reasonable approach. Conversely, in locally advanced disease and in tumors with ISUP grade ≥3, both focal and extensive PSMs were associated with a substantially increased risk of biochemical recurrence, supporting closer follow-up and consideration of adjuvant treatment.

## 1. Introduction

Positive surgical margins (PSMs) are a frequent adverse finding after radical prostatectomy and are associated with an increased risk of biochemical recurrence (BCR). However, PSMs are not all the same: a focal ink-touching margin may reflect a limited capsular incision with minimal or no residual burden, whereas longer or multifocal margins are more likely to indicate clinically meaningful residual tumour. Distinguishing these scenarios is increasingly relevant for postoperative counselling, surveillance intensity, and timing of salvage treatment. Accordingly, several studies suggest that PSM “subclassification”—by extent/length, focality, and ideally tumour grade at the margin—adds prognostic information beyond the simple positive/negative report, although its routine clinical utility remains debated and has not been consistently incorporated into trial designs [1].

A 3 mm threshold has emerged as a pragmatic cut-off: focal/unifocal PSMs < 3 mm may have limited oncologic impact in otherwise favourable pathology, while PSMs ≥ 3 mm and/or multifocality are more often associated with higher BCR risk and may justify closer follow-up or earlier intervention [2]. Importantly, the effect of a focal margin likely depends on tumour biology; in aggressive disease, even limited margin involvement may behave like an unfavourable PSM, whereas in indolent tumours it can approximate negative margins [3].

Despite this rationale, real-world evidence quantifying how the prognostic meaning of focal versus extensive PSMs changes across pathological stage and grade remains limited [4]. The aim of this study was to evaluate the association between margin status (negative, focal, extensive) and biochemical outcomes after robot-assisted radical prostatectomy (RARP), focusing on effect modification by pathological stage and grade.

## 2. Patients and Methods

We conducted a retrospective cohort study including consecutive patients undergoing RARP for prostate cancer (PCa) at a tertiary referral centre between 2017 and 2023. Patients were included if complete perioperative, pathological, and follow-up data were available. According to Agenzia Italiana del Farmaco (AIFA) guidance for observational studies, formal ethics committee approval was not required. RARP was performed according to standard, anterior approach as previously described [5]. Pelvic lymph node dissection was performed based on preoperative risk assessment and surgeon judgement, and nodal status was recorded as pN0/pN1 when lymph nodes were removed.

RARP specimens were processed according to institutional routine. A PSM was defined as the presence of tumour cells in contact with the inked surface of the specimen. PSMs were subclassified according to their linear extent, distinguishing between focal (fPSM) if ≤3 mm and extensive (ePSM) if >3 mm. We defined PSM length as the maximum linear extent of a single positive-margin focus; multifocality and anatomical location of PSMs were not routinely captured in our database. Pathological variables included ISUP grade group, pathological stage (pT2, pT3a, pT3b), and nodal status (pN0/pN1). Postoperative follow-up consisted of periodic PSA testing and clinical visits according to institutional practice. Outcomes were defined as biochemical persistence (BCP), defined as PSA ≥ 0.1 ng/mL within 6 months after surgery, and biochemical recurrence (BCR), defined as PSA ≥ 0.2 ng/mL 6 months after surgery.

### Statistical Analyses

Baseline characteristics and pathological features were compared using chi-square tests for categorical variables and Kruskal–Wallis tests for continuous variables, as appropriate. BCR-free survival (BCRFS) was estimated using the Kaplan–Meier method and compared with log-rank tests. Cox proportional hazards regression was used to identify predictors of BCR. Two multivariable Cox models were built: Model 1 included preoperative/clinical variables (age, PSA, MRI tumour diameter) plus key pathological variables to reflect a ‘clinical–pathological’ setting; Model 2 was pathology-focused and included only surgical–pathological variables to assess the independent contribution of PSMs in the postoperative pathology context and to reduce collinearity/overadjustment. Postoperative adjuvant/salvage radiotherapy and androgen deprivation therapy were not modelled as time-dependent covariates, given their strong dependence on pathological findings (confounding by indication); this is addressed as a limitation. Statistical significance was set at *p* < 0.05. Analyses were performed using SPSS (v29, IBM, Armonk, NY, USA).

## 3. Results

The cohort included 1258 men (median follow-up 39 months, IQR 20–62). Baseline patients’ characteristics are shown in Table 1. Overall, 238 patients (18.9%) had PSMs—147 (11.7%) focal and 91 (7.2%) extensive—while 1020 (81.2%) had negative margins. Preoperative risk profiles differed across margin groups. Median PSA was higher in the extensive PSM group (9.6 ng/mL) compared with the focal PSM group (8.05 ng/mL) and negative margins (6.7 ng/mL) (*p* < 0.001). Clinical nodal positivity (cN1) and mpMRI-suspicious nodal status were more frequent among men with extensive PSMs, consistent with a higher baseline risk profile. The higher rate of positive DRE in the extensive PSM group likely reflects a higher baseline local tumour burden in this subgroup.

Surgical and oncological outcomes are shown in Table 2. Margin status strongly correlated with adverse pathology. Extensive PSMs were higher in advanced stage and higher grade: pT3b accounted for 41.8% of extensive PSMs versus 25.9% of focal PSMs and 9.6% of negative margins (*p* < 0.001). Similarly, ISUP grade group 4–5 was more frequent in the extensive PSM group (approximately 46% combined) than in focal PSMs and negative margins (*p* < 0.001). Among those who underwent pelvic lymphadenectomy, nodal positivity (pN1) was also highest among extensive PSMs (34.6%) compared with focal PSMs (29.5%) and negative margins (12.5%) (*p* < 0.001).

The use of postoperative therapies reflected the different risk strata: adjuvant radiotherapy was administered to 26.4% of men with extensive PSMs compared with 17.9% with focal PSMs and 4.4% with negative margins (*p* < 0.001). Overall, BCR occurred in 255 patients (20.3%), with a gradient by margin subgroup: 16.0% in negative margins, 32.7% in focal PSMs, and 48.4% in extensive PSMs (*p* < 0.001). Biochemical persistence showed a similar stepwise pattern (4.9%, 15.0%, and 31.9%, respectively; *p* < 0.001).

### 3.1. Kaplan–Meier Analyses: Effect Modification by Grade and Stage

**Figure 1:**
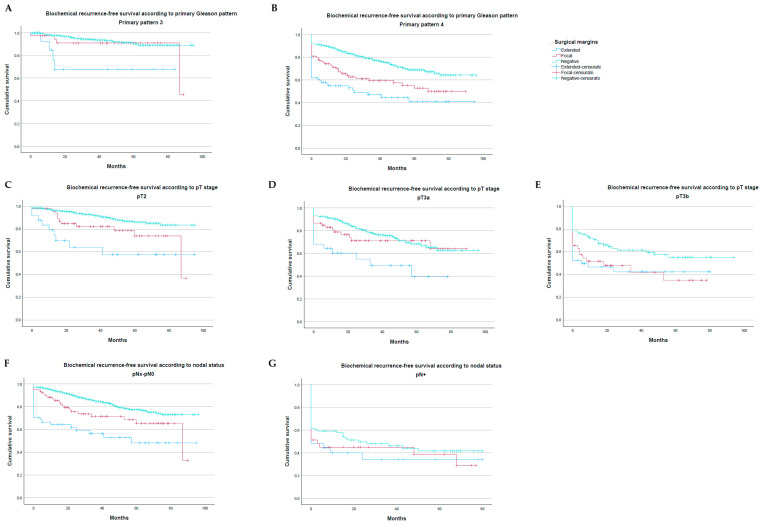
Survival curves showing the prognostic significance of focal and extensive PSMs across different stages and grades. (**A**) Primary Gleason pattern 3; (**B**) Primary Gleason pattern 4; (**C**) pT2; (**D**) pT3a; (**E**) pT3b; (**F**) pNx-pN0; (**G**) pN+.

Kaplan–Meier curves demonstrated that the prognostic meaning of focal PSMs was not uniform, but varied by tumour biology. In men with more favourable disease features such as lower grade and organ-confined tumours, focal PSM curves approximated those of negative margins, whereas in higher-risk settings (higher primary Gleason pattern/grade groups, extraprostatic extension and seminal vesicle invasion, nodal positivity), focal PSM curves shifted toward the behaviour of extensive PSMs (Figure 1). Biochemical recurrence-free survival varied significantly according to margin status (focal versus extensive versus negative) in combination with different pathological stage and grade (Supplementary Table S1). In pT2 tumours or primary Gleason pattern 3, focal PSMs showed 5-year BCRFS of 88–90%, very similar to negative margins (91–93%) and clearly better than extensive PSMs (~72%). In pT3 tumours or primary Gleason pattern 4, focal PSMs showed 5-year BCRFS of ~55–60%, similar to extensive PSMs (~40–50%) and significantly worse than negative margins (78% in pT3a and 65% in pT3b, *p* < 0.01). In *n* + 5-year BCRFS was similarly poor in negative (~45%), focal (~42%), and extensive margins (~40%, *p* = 0.455).

### 3.2. Cox Regression Analyses: Predictors of BCR

In Cox regression (Table 3), extensive PSMs were consistently associated with increased BCR risk. In the clinical–pathological multivariable model, extensive PSMs remained an independent predictor of BCR (HR 2.22, 95% CI 1.35–3.64; *p* = 0.002), whereas focal PSMs was not significant (HR 1.13, 95% CI 0.73–1.76; *p* = 0.58). Nodal positivity showed the strongest association (HR 3.40, 95% CI 2.11–5.48; *p* < 0.001). In the pathology-focused multivariable model, both focal and extensive PSMs retained independent associations with BCR (focal HR 1.44, *p* = 0.04; extensive HR 1.94, *p* < 0.001), alongside grade group, pT stage, and nodal status.

## 4. Discussion

In this large contemporary cohort of men who underwent RARP, we found that the prognostic meaning of a positive surgical margin (PSM) is not uniform but strongly depends on tumour biology and pathological extent. Specifically, focal PSMs behaved similarly to negative margins in favourable disease (pT2 tumours and primary Gleason pattern 3), whereas in adverse disease (pT3 tumours and primary Gleason pattern 4) focal PSMs approximated the risk profile of extensive PSMs. As expected, in node-positive patients, margin status did not meaningfully stratify BCRFS, suggesting that once systemic dissemination is established, local margin characteristics become less relevant for biochemical control. We highlight that nowadays the indication to adjuvant treatment after RARP is rarely given, preferring an early salvage treatment in case of BCR, as confirmed by the low percentages of adjuvant RT/ADT administered in this series. Therefore, adjuvant treatments had a little impact on BCRFS in this study.

The clinical significance of PSMs after radical prostatectomy remains debated because PSMs may represent different biological scenarios: true residual disease, capsular incision in organ-confined tumours, artefacts related to specimen processing, or a surrogate of more aggressive local extension. Our data support the concept that PSMs should be interpreted as an interaction between local surgical/pathological findings and tumour aggressiveness. In low-risk settings, focal involvement likely reflects minimal or even no residual burden, leading to outcomes close to those of negative margins; conversely, when grade and/or stage are unfavourable, even limited margin involvement may indicate biologically aggressive disease or microscopic extension beyond the resection plane, translating into a recurrence risk comparable to that of extensive PSMs.

Our findings are consistent with the increasing evidence that stage and grade are the dominant determinants of BCR after prostatectomy and may modulate the marginal impact of PSMs. Carbonara et al. reported that ISUP grade and tumour stage were the most important predictors of BCR after RARP; notably, PSMs predicted BCR in pT2 disease, whereas in pT3 disease, the key driver was pT3b, and margin parameters were not independently predictive [6].

This aligns closely with our observation that focal PSMs “shift” prognostic behaviour across pathological strata. Moreover, prediction tools increasingly combine multiple pathological and biochemical factors rather than relying on PSM status alone. In a recent nomogram, Blas et al. incorporated pathological Gleason score, pathological T stage, PSM, PSA ≥ 0.05 ng/mL at one year, and lymph node involvement to predict BCR-free survival, underscoring that margins contribute to risk only within a broader multivariable framework [7].

Another key point emerging from the literature is that not all margin information is equivalent, and “PSM yes/no” may be too crude. Several studies suggest that more detailed margin pathology—particularly Gleason grade/grade group at the margin and cumulative margin length—can refine prognostication. Remmers et al. showed that adding cribriform growth, grade group at the PSM, and cumulative PSM length modestly improved BCR prediction compared to a baseline model including PSA, grade group in the primary tumour, pT stage, pN stage, and PSM presence; importantly, men with grade group 1 at the margin did not have higher BCR risk than those with negative margins, supporting the concept that a subset of “favourable” PSMs may be clinically indolent [8].

Similar messages have been emphasized across a contemporary review of the literature addressing the heterogeneity of margin parameters and their incremental value beyond established adverse features [9].

Beyond predicting BCR, the clinical impact of BCR itself has been increasingly scrutinized, because biochemical relapse is frequent but does not uniformly translate into metastasis or prostate cancer–specific mortality. In a large population-based study, Falagario et al. found that BCR after primary treatment was common yet had a limited association with cancer-related mortality, highlighting the need for improved recurrence definitions and risk stratification [10]. Complementarily, very late events may be rare and clinically limited in selected subgroups: Hoeh et al. reported low rates of late BCR and very low metastatic progression among men disease-free for ≥10 years after prostatectomy, particularly for favourable pathological categories [11].

From a practical standpoint, our results may help tailor the follow-up intensity of our patients and refine decision-making regarding adjuvant versus early salvage treatments [12,13]. In pT3 tumours and/or primary Gleason pattern 4, focal margins carried a recurrence risk similar to extensive margins, supporting the rationale for intensified monitoring and an earlier threshold for multimodal approaches in the presence of additional adverse features. Finally, in pN+ disease, margin status did not stratify outcomes, indicating that systemic disease burden likely dominates the recurrence trajectory and that management decisions should prioritize nodal status and systemic risk factors.

This study has several limitations. Its retrospective design may introduce selection bias, and follow-up length may not capture late recurrences. Most importantly, we did not perform a centralized pathological review and did not assess detailed margin pathology such as grade group at the margin, PSM location, or precise cumulative margin length, factors that have been shown to refine BCR risk and may identify “favourable” PSM subsets [8]. The absence of grading at the margin is particularly relevant given emerging evidence that low-grade tumour at the margin may carry minimal incremental risk compared with negative margins [9]. On the other hand, our data come from a large and homogeneous series of RARP and could serve as a benchmark for future studies integrating grade at margin and quantitative extent, ideally with central review.

## 5. Conclusions

The prognostic role of focal PSMs after radical prostatectomy is stage- and grade-dependent: it may be clinically negligible in favourable disease but becomes comparable to extended margins in adverse pathology, while losing discriminatory value in node-positive patients. Incorporating this interaction into postoperative counselling may help tailor follow-up intensity, reassuring the patient in the case of a focal PSM with favourable pathological features, while suggesting early intervention for patients with a PSM and biologically aggressive disease.

## Figures and Tables

**Table 1:** Baseline patient characteristics.

Characteristic	Overall	Focal PSM	Extensive PSM	Negative Surgical Margins	*p*
Number of patients	1258	147 (11.7%)	91 (7.2%)	1020 (81.2%)	-
Age at surgery, yr, median (IQR)	68 (63–73)	69 (64–73)	68 (63–74)	68 (63–73)	0.76
Preoperative PSA, ng/mL, median (IQR)	6.9 (5.1–10.0)	8.0 (5.8–13.6)	9.6 (6.0–17.0)	6.7 (5.0–9.4)	<0.001
Prostate volume, cc, median (IQR)	41.0 (30.5–55.3)	40.1 (30.0–53.0)	42.0 (29.0–56.7)	41.0 (30.6–56.0)	0.58
Positive DRE, *n* (%)	602 (47.9)	74 (50.3)	55 (60.4)	473 (46.4)	0.03
Extracapsular extension at MRI, *n* (%)	319 (28.1)	30 (24.0)	27 (35.5)	262 (28.1)	0.21
Seminal vesicle involvement at MRI, *n* (%)	191 (16.8)	18 (14.4)	18 (23.7)	155 (16.6)	0.21
cN+ at preoperative imaging, *n* (%)	54 (4.8)	9 (7.2)	10 (13.2)	35 (3.7)	<0.001
Biopsy ISUP grade, *n* (%)					<0.001
-1	100 (7.9)	10 (6.8)	9 (9.9)	81 (7.9)	
-2	522 (41.5)	52 (35.4)	20 (22)	450 (44.1)	
-3	377 (30.0)	46 (31.3)	28 (30.8)	303 (29.7)	
-4	181 (14.4)	32 (21.8)	14 (15.4)	135 (13.2)	
-5	78 (6.2)	7 (4.8)	20 (22)	51 (5.0)	
Neoadjuvant therapy, *n* (%)	48 (3.8)	10 (6.8)	7 (7.8)	31 (3.1)	0.01

Legend: DRE: digital rectal examination; ISUP: International Society of Urological Pathology; IQR: interquartile range; MRI: magnetic resonance imaging.

**Table 2:** Surgical and oncological outcomes and postoperative treatments.

Characteristic	Overall	Focal PSM	Extensive PSM	Negative Surgical Margins	*p*
Number of patients	1258	147 (11.7%)	91 (7.2%)	1020 (81.2%)	-
Nerve sparing, *n* (%)					<0.001
-Monolateral	443 (35.2)	42 (28.6)	22 (24.2)	379 (37.2)	
-Bilateral	424 (33.7)	37 (25.2)	22 (24.2)	365 (35.8)	
Pathologic ISUP grade, *n* (%)					<0.001
-1	10 (0.8)	2 (1.4)	0 (0)	8 (0.8)	
-2	485 (38.6)	35 (23.8)	15 (16.5)	435 (42.7)	
-3	521 (41.4)	69 (46.9)	34 (37.4)	418 (41.0)	
-4	141 (11.2)	24 (16.3)	16 (17.6)	101 (9.9)	
-5	100 (8.0)	17 (11.6)	26 (28.6)	57 (5.6)	
Pathologic stage, *n* (%)					<0.001
-pT2	709 (56.4)	50 (34.0)	25 (27.5)	634 (62.2)	
-pT3a	375 (29.8)	59 (40.1)	28 (30.8)	288 (28.2)	
-pT3b	174 (13.8)	38 (25.9)	38 (41.8)	98 (9.6)	
Pelvic lymph node dissection, *n* (%)	866 (68.8)	112 (76.2)	78 (85.7)	676 (66.2)	<0.001
pN1, *n* (%) *	146 (16.6)	33 (29.5)	27 (34.6)	86 (12.5)	<0.001
Nodes removed, *n*, median (IQR) *	20 (14–26)	20 (15–27)	20 (14–27)	20 (14–25)	0.53
Nodes positive, *n*, median (IQR) *	0 (0–0)	0 (0–1)	0 (0–1)	0 (0–0)	<0.001
Oncological outcomes					
Biochemical persistence, *n* (%)	101 (8.0)	22 (15.0)	29 (31.9)	50 (4.9)	<0.001
Biochemical recurrence, *n* (%)	255 (20.3)	48 (32.7)	44 (48.4)	163 (16.0)	<0.001
Biochemical recurrence-free survival, mean, months (95% CI)	74.9 (72.5–77.2)	60.6 (53.9–67.2)	48.5 (38.7–58.2)	79.2 (76.8–81.5)	<0.001
Cancer-specific mortality, *n* (%)	13 (1.0)	6 (4.1)	3 (3.3)	4 (0.4)	<0.001
Cancer specific survival, mean, months (95%CI)	94.6 (93.7–95.5)	86.0 (82.8–89.1)	91.4 (87.4–95.4)	95.2 (94.3–96.1)	<0.001
Follow-up, months, median (IQR)	39 (17–60)	39 (19–68)	41 (17–63)	39 (16–59)	0.92
Postoperative treatments					
Adjuvant RT, *n* (%)	95 (7.6)	26 (17.9)	24 (26.4)	45 (4.4)	<0.001
Adjuvant ADT, *n* (%)	110 (8.8)	23 (16.0)	24 (26.7)	63 (6.2)	
Salvage RT, *n* (%)	120 (9.6)	24 (16.6)	17 (18.7)	79 (7.7)	<0.001
Salvage ADT, *n* (%)	63 (5.0)	13 (9.0)	14 (15.6)	36 (3.5)	

Legend: ADT: antiandrogen therapy; IQR: interquartile range; ISUP: International Society of Urological Pathology; RT: radiotherapy. * Nodes removed/positive were calculated among 877 patients who received pelvic lymph node dissection.

**Table 3:** Predictors of biochemical recurrence in Cox regression survival analysis.

	Univariate Analysis		Multivariate Analysis Model 1		Multivariate Analysis Model 2	
Variable	HR (95%CI)	*p*	HR (95%CI)	*p*	HR (95%CI)	*p*
Age	1.02 (1.00–1.04)	0.02	1.02 (1.00–1.05)	0.053	-	-
PSA	1.01 (1.01–1.01)	<0.001	1.00 (0.99–1.02)	0.16	-	-
ISUP grade						
-<3	Ref.		Ref.		Ref.	
-≥3	4.39 (3.08–6.24)	<0.001	1.46 (0.84–2.56)	0.17	1.70 (1.04–2.77)	0.03
pT stage						
-pT2	Ref.		Ref.		Ref.	
-pT3a	2.66 (1.97–3.60)	<0.001	1.46 (0.84–2.56)	0.07	1.92 (1.35–2.74)	<0.001
-pT3b	5.65 (4.12–7.76)	<0.001	1.41 (0.83–2.40)	0.20	2.20 (1.44–3.34)	<0.001
PSM						
-negative	Ref.		Ref.		Ref.	
-focal	2.22 (1.61–3.07)	<0.001	1.13 (0.72–1.78)	0.58	1.44 (1.01–2.04)	0.04
-extensive	3.86 (2.76–5.39)	<0.001	2.22 (1.35–3.65)	0.002	1.94 (1.34–2.81)	<0.001
pN stage						
-pNx-N0	Ref.		Ref.		Ref.	
-pN1	4.03 (3.06–5.30)	<0.001	3.40 (2.23–5.19)	<0.001	2.45 (1.78–3.37)	<0.001
Largest tumour diameter at MRI	1.06 (1.04–1.07)	<0.001	1.01 (0.99–1.04)	0.14	-	-

Legend: HR: hazard ratio; PSM: positive surgical margin.

## Data Availability

The data presented in this study are available on request from the corresponding author.

## Supplementary Materials

The following supporting information can be downloaded at https://www.mdpi.com/article/10.3390/cancers18071123/s1, Table S1. Biochemical recurrence-free survival according to margin status combined with pathological stage and grade.
