# Supplementary material for: Pathological Stage and Grade Determine the Impact of Focal Versus Extensive Positive Surgical Margins After Radical Prostatectomy
Source: Cancers (Basel). 2026 Mar 31;18(7):1123. doi: 10.3390/cancers18071123 (PMC13072274; doi:10.3390/cancers18071123)
Supplement: Supplementary file 1 [file cancers-18-01123-s001.zip › cancers-4137465-supplementary.pdf]

Table S1. Biochemical recurrence-free survival according to margin status combined with pathological stage and grade.

Characteristics		BCRFS. mean. months (95%CI)	*p* (within group)	*p* (adjusted)
pT stage	-	-	-	<0.001
	pT2		<0.001	
	- Focal PSM	72.7 (63.9-81.5)		
	- Extended PSM	35.4 (43.3-78.2)		
	- Negative surgical margins	85.5 (83.1-87.9)		
	pT3a		0.002	
	- Focal PSM	63.9 (53.6-74.3)		
	- Extended PSM	40.2 (26.5-54.0)		
	- Negative surgical margins	71.6 (66.7-76.4)		
	pT3b		0.039	
	- Focal PSM	34.5 (22.4-46.6)		
	- Extended PSM	35.4 (22.7-48.1)		
	- Negative surgical margins	57.4 (48.4-66.4)		
ISUP grade	-	-	-	<0.001
	ISUP grade < 3		0.003	
	- Focal PSM	80.9 (73.0-88.7)		
	- Extended PSM	60.5 (41.4-79.5)		
	- Negative surgical margins	88.2 (85.7-90.6)		
	ISUP grade ≥ 3		<0.001	
	- Focal PSM	53.8 (45.7-61.9)		
	- Extended PSM	44.5 (33.9-55.1)		
	- Negative surgical margins	72.0 (68.6-75.5)		
pN stage	-	-	-	<0.001
	pN+		0.455	
	- Focal PSM	32.2 (19.7-44.7)		
	- Extended PSM	29.4 (14.9-43.9)		
	- Negative surgical margins	38.2 (30.0-46.4)		
	pNx-pN0		<0.001	
	- Focal PSM	65.2 (56.7-73.7)		
	- Extended PSM	52.6 (39.9-65.2)		
	- Negative surgical margins	79.3 (76.4-82.3)		
